# Supplementary material for: Investigation of Fiber–Matrix Interface Strength via Single-Fiber Pull-Out Test in 3D-Printed Thermoset Composites: A Simplified Methodology
Source: Materials (Basel). 2024 May 18;17(10):2433. doi: 10.3390/ma17102433 (PMC11122960; doi:10.3390/ma17102433)
Supplement: Supplementary file 1 [file materials-17-02433-s001.zip › materials-2955002-supplementary.pdf]

Supplementary Material

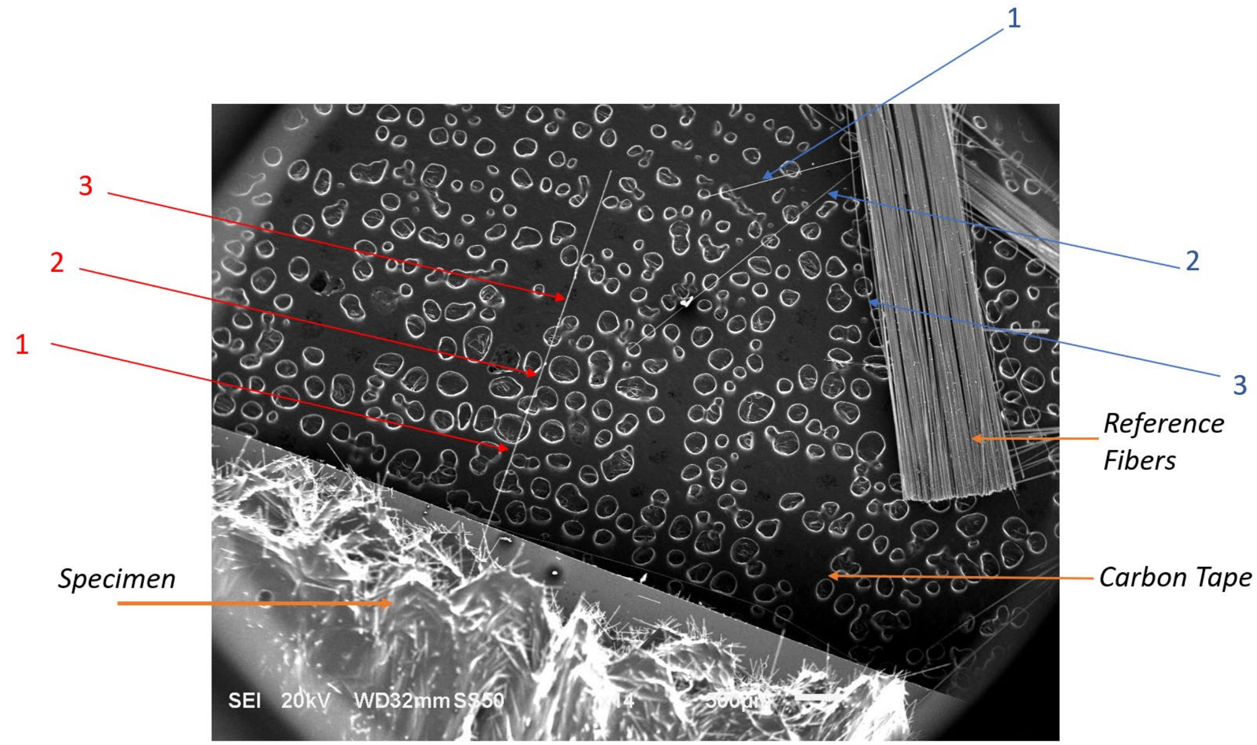

Figure S1. SEM picture of the EDS analysis including pull-out specimen and reference fibers.

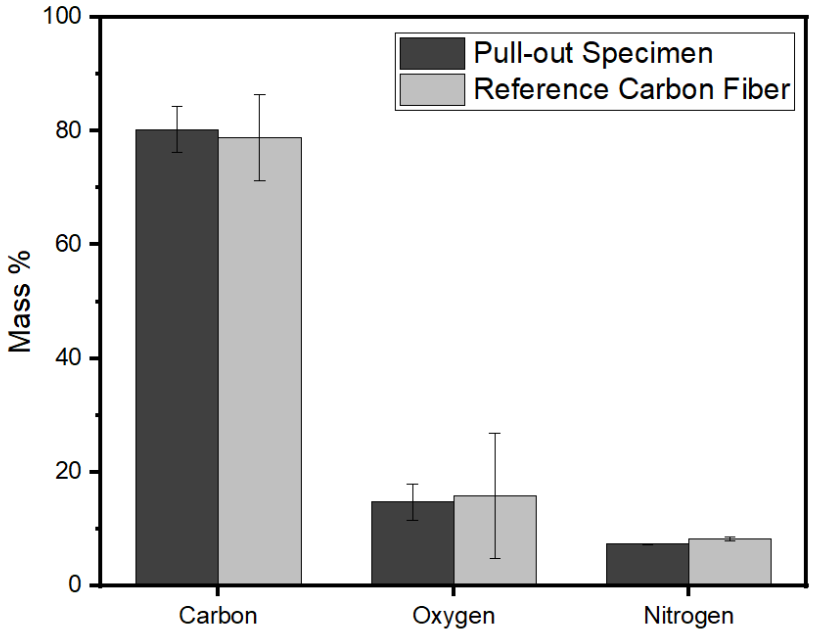

Figure S2. Average mass% comparisons of C, O and N atoms on the fibers.

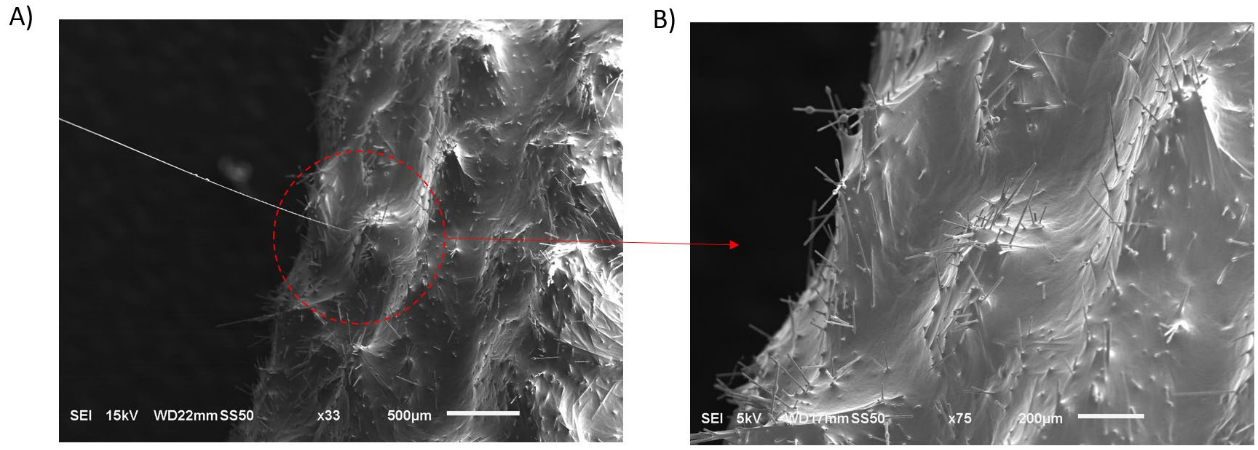

Figure S3. SEM image of the pullout sample A) before the test, B) after the test.
